# Phenotyping of the Visceral Adipose Tissue Using Dual Energy X-ray Absorptiometry (DXA) and Magnetic Resonance Imaging (MRI) in Pigs

**DOI:** 10.3390/ani10071165

**Published:** 2020-07-09

**Authors:** Anna C. Weigand, Helen Schweizer, Deise Aline Knob, Armin M. Scholz

**Affiliations:** 1 Livestock Center Oberschleissheim, Veterinary Faculty of the Ludwig-Maximilians-University Munich, 85764 Oberschleissheim, Germany; Helen.Schweizer@lvg.vetmed.uni-muenchen.de (H.S.); Armin.Scholz@lvg.vetmed.uni-muenchen.de (A.M.S.); 2 Centro de Ciências Agroveterinárias, Universidade do Estado de Santa Catarina, Lages CEP 88.520-000, Brazil; deisealinek@gmail.com

**Keywords:** dual energy X-ray, MRI, CoreScan, visceral adipose tissue, pig

## Abstract

**Simple Summary:**

Fat depots in the abdomen and around the organs, which are called visceral adipose tissue, play an important role in the field of obesity-associated diseases in humans and for pork production as well. Magnetic resonance imaging—as reference method—and a special X-ray technique called dual energy X-ray absorptiometry were used to measure the visceral adipose tissue in 120 pigs in order to analyze the accuracy of a special X-ray software algorithm (the “CoreScan” mode), and to study sex or crossbreed-related effects. The “CoreScan” mode overestimates the amount of visceral adipose tissue in comparison with magnetic resonance imaging, while castrated males tend to accumulate more visceral adipose tissue than females, and the first crossbred generation deposits more fat than the second generation.

**Abstract:**

The objective of this study was to phenotype visceral adipose tissue (VAT) in pigs. In this context, the ability to detect VAT by using the DXA CoreScan mode within the enCORE software, version 17 (GE Healthcare) was evaluated in comparison with MRI measurements (Siemens Magnetom C!) of the same body region. A number of 120 crossbred pigs of the F1 and F2 generation, with the parental breeds Large White, Landrace, Piétrain, and Duroc, were examined at an age of 150 days. A whole-body scan in two different modes (“thick”, “standard”) was carried out by a GE Lunar iDXA scanner. Very strong relationships (R^2^ = 0.95, RMSE = 175 cm^3^) were found for VAT between the two DXA modes. The comparison of VAT measured by MRI and DXA shows high linear relationships (“thick”: R^2^ = 0.76, RMSE = 399.25 cm^3^/“standard”: R^2^ = 0.71, RMSE = 443.42 cm^3^), but is biased, according to the Bland–Altman analysis. A variance analysis of VAT shows significant differences for both DXA modes and for MRI between male and female pigs, as well as between F1 and F2. In conclusion, DXA “CoreScan” has the ability to estimate VAT in pigs with a close relationship to MRI but needs bias correction.

## 1. Introduction

Cardiovascular disease (CVD) is the most common cause of death in Germany and the entire world in the last few years. In 2016, 17.9 million people worldwide died from CVDs, which constituted 31% of all deaths. In Germany, the percentage was even higher [1,2]. The most important cardiovascular diseases are coronary heart disease (CHD), manifested by myocardial infarction and heart failure, and cerebrovascular disease, including stroke [1,3,4,5,6]. Cardiometabolic risk factors, such as hypertension, dyslipidemia, and metabolic syndrome, are associated with visceral adipose tissue (VAT) [7,8].

Against the backdrop of high mortality, resulting from CVD, the need for precise measurement methods of VAT is immense. With their CoreScan mode, GE Healthcare offers the possibility of determining visceral adipose tissue by using dual energy X-ray absorptiometry (DXA). This study examined the ability of CoreScan to precisely quantify the content of visceral adipose tissue in pigs compared to the results of magnet resonance imaging (MRI).

The pig plays an increasing role as a model in human research due to the similarity in anatomic and physiologic characteristics involving the cardiovascular, urinary, integumentary, and digestive systems [9,10,11]. Furthermore, the interest in investigating obesity-related genes in pigs is explained by the fact that the porcine genome is even more similar to humans than the genome of mice [12].

In addition, new insights into the fat depot metabolism are important for the commercial rearing of pigs. The production of high-quality meat at low costs is still one of the primary objectives. The combination of exact phenotypic data with genome data will provide further knowledge of the regulation of body composition [13,14,15].

The aim of this study was to phenotype the visceral adipose tissue in pigs using MRI, and to evaluate the ability of the DXA CoreScan mode to be a fast, simple, and low-cost option to quantify the volume of visceral adipose tissue, compared to an MRI reference examination.

## 2. Materials and Methods

### 2.1. Animals

A number of 120 crossbred pigs of the first and second filial generation (F1, F2), with the parental breeds Large White, Landrace, Piétrain, and Duroc, were examined. The number of pigs of the F1 crossbreed, called Multi-hybrid-F1 (MHF1) and the F2 crossbreed, Multi-hybrid-F2 (MHF2), and also the number of females and castrated males, was supposed to be balanced, as shown in Table 1. All pigs were born and raised at the Livestock Center Oberschleissheim and were treated like the other pigs in the facility. Males were castrated on the third day of life. After a suckling period of four weeks the piglets were weaned and moved to a nursery deck, where they stayed in groups of 15–20 animals. With a weight above 25 kg, the pigs were housed in an outdoor-climate barn with various climatic and flooring areas in groups of 30–34 animals. The feeding area is enlarged and consists of concrete-slatted flooring; the rest is deeply bedded with straw and contains a hooded area with a special (warmer) microclimate during the winter season. The feed is individually allocated via an automatic feeder (Double-Fitmix, Mannebeck). All pigs received the same food throughout the experiment.

This study was conducted at the Livestock Center Oberschleissheim of the Veterinary Faculty of the Ludwig-Maximilians-Universität in Munich with the approval of the District Government of Upper Bavaria (No. 55.2-1-54-2532.0-81-2016).

### 2.2. Test Procedure

The pigs were studied at an average age of 147 days and an average weight of 91.6 kg, as shown in Table 1. Animals were selected by their genetic origin and randomly within the litters. For the scan by MRI and DXA, sedation was needed to achieve profound results by reducing the movement of the individual. One day before the scan, the animals were moved to a barn nearby the MRI unit and housed in groups with concrete flooring and straw bedding. To reduce the risk of an anesthetic accident, the feed was deprived of the experimental pigs for 16 h. Water was available ad libitum. At the day of scan, the health status of each animal was checked, and the weight was measured with a mechanic livestock scale for the exact dosage of the anesthetic. Azaperon (2 mg/kg bw, Stresnil^®^, Elanco) and ketamine (10–15 mg/kg bw, Ursotamin^®^, Serumwerk Bernburg) were used for the anesthesia by intramuscular injection. A catheter was inserted into the vena auricularis to be able to administer additional ketamine, if necessary. The monitoring of anesthesia was carried out via a scoresheet by recording different parameters, such as heart rate, respiratory frequency, and capillary filling time.

#### 2.2.1. Magnetic Resonance Imaging

**Figure 1:**
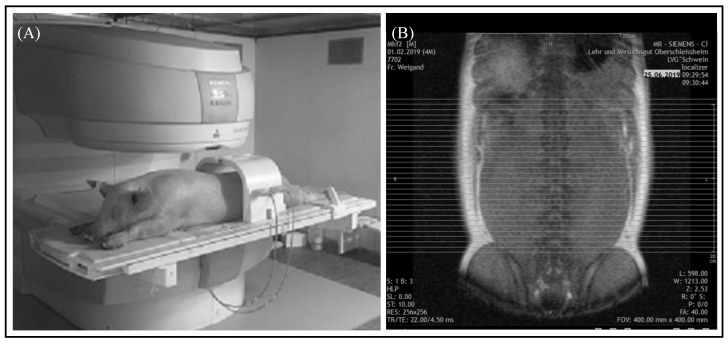
MRI examination of the abdomen. (**A**) Position of the pig for the examination of the abdomen by a Siemens Magnetom C! system. (**B**) Localizer of the “ViscFat-Sequence” with defined area encompassing visceral adipose tissue. Each line represents an axial sectional image of the abdomen starting at the origin of the last rib to the top of the iliac crest.

An open low-field MRI system (Siemens Magnetom C!) with a magnetic field strength of 0.35 Tesla was used for the scans. A fully automated quality control was executed by the Siemens Magnetom C! system at each restart of the device [16]. The animals were placed in a prone position with their forelimbs flexed and hind limbs extended to enable the examination of both body halves at the same time. Anatomic landmarks were used for the positioning in order to acquire reproducible and comparable images for the same body region. In the area of the abdomen, the origin of the last rib and in the section of the pelvis, the top of the iliac crest were taken as a guide, as shown in Figure 1A,B.

Two T1-weighted spin echo sequences in axial acquisition per animal were applied to detect the visceral adipose tissue. The first sequence, called “ViscFat sequence”, covered most of the abdomen, as shown in Figure 1, and the second sequence, “Ham sequence” included the remaining part of the abdomen and the region of the pelvis. In larger animals the first sequence was not sufficient to cover the whole region of interest; therefore, selected slices of the “Ham sequence” were additionally used for the evaluation. The protocols of MRI settings are shown in Table 2.

#### 2.2.2. Dual Energy X-ray Absorptiometry

**Figure 2:**
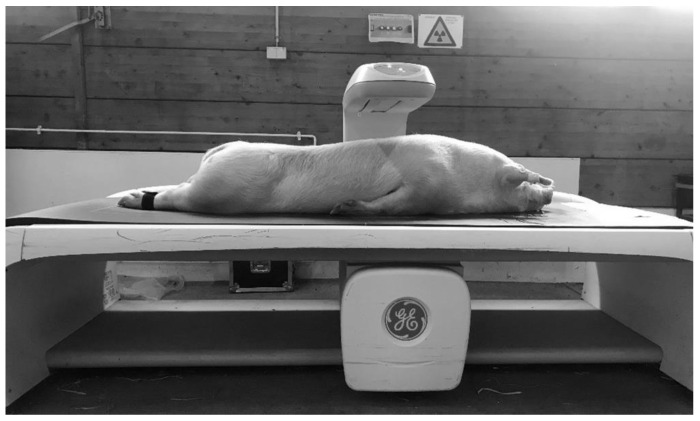
DXA examination.

After the MRI scan, the body composition of the pigs was measured by DXA, as shown in Figure 2. Generally, the measurement of DXA bone mineral and soft tissue amounts (divided into fat and lean) is based on the X-ray attenuation coefficient (R value). The R value (not shown in the *i*DXA system) is derived pixel wise from the quotient of X-ray photons of the high and low X-ray energy level, having passed the object. Low R values define soft tissue and high R values bone mineral. Within soft tissue, fat tissue is characterized by the lowest R values [15,17]. The GE Lunar *i*DXA scanner provides a special mode for the whole body scan, based on the body constitution. For this study, the two different modes—“thick” and “standard”—were used to obtain information about the amount and percentage of fat and lean tissue mass, bone mineral content, and bone mineral density. The whole body scan in “thick” mode takes 13.45 min with an absorbed dose of 6 µGy, and in “standard”mode this was 7.45 min with a dose of 3 µGy. In addition, GE Healthcare, with the CoreScan mode within the enCORE software version 17, offers the ability to determine the mass (g) and volume (cm^3^) of the visceral adipose tissue in adults. The *i*DXA system measures the whole fat in the android region. The subcutaneous adipose tissue is detected by a hidden geometric calculation and subtracted from the total adipose tissue to obtain VAT [18]. The algorithm works with the detection of two parameters. The two parameters are: (1) the width of the SAT layer on lateral aspects of the body, which is represented by the fat between the skin line and the outer abdominal wall on both sides of the image; (2) the anterior–posterior thickness of the abdomen, which can be attained using the DXA tissue attenuation image [19]. The algorithms for the determination of VAT are described in detail in the US and world patents: US9179884 (https://patents.google.com/patent/US9179884); US9254101 (https://patents.google.com/patent/US9254101); WO2012092533Al (https://patents.google.com/patent/WO2012092533A1/en), from GENERAL ELECTRIC COMPANY, Schenectady, NY (US). Before the examination, a quality control of the *i*DXA system was executed, according to the standard procedures, once a day [20]. The pig was bedded in a prone position with forelimbs and hind limbs extended to simulate the suggested position for whole body DXA scans in humans. Foam cushions were used to create a gap between the abdominal wall and the forelimbs to prevent overlays. The hind limbs were tied together.

### 2.3. Data Analysis

#### 2.3.1. MRI Evaluation

**Figure 3:**
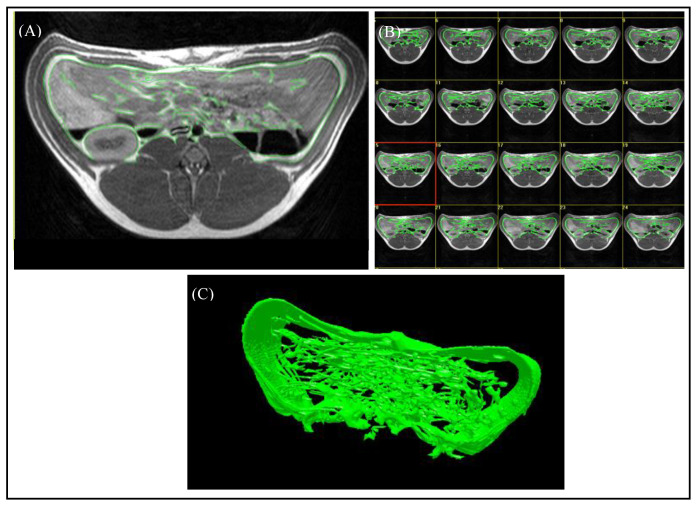
Evaluation of MR images using the Able 3D-Doctor^®^ software. (**A**) T1-weighted axial MR image with green boundaries, including the visceral adipose tissue. (**B**) Analysis of all slices in the defined body region. (**C**) Reconstruction of a 3D-model from the selected boundaries of VAT.

For the evaluation of the magnetic resonance images, the “synedra” View Personal software (Version 16.0.0.3, synedra information technologies GmbH, Innsbruck, Austria) and the Able 3D-Doctor^®^ software (Release 4.0; Able Software Corp., Lexington, MA, USA) were used. The region containing VAT was defined as the area between the origin of the last rib and the top of the iliac crest. The number of slices of the “ViscFat” and “Ham” sequences, which cover the region of interest (ROI), was determined with the help of “synedra”, as shown in Figure 1B. For the evaluation of the VAT volume, the regions of VAT were defined manually for every single slice, including the fat depots beneath the abdominal wall, around the kidneys, and in the abdominal cavity, as shown in Figure 3. As the peritoneum is often difficult to discern with the use of MRI, in this study, VAT was defined as the intra-abdominal adipose tissue; no distinction between intra- and extraperitoneal adipose tissue was assigned. Consequently, the Able 3D-Doctor^®^ software provides an option to calculate the volume of the defined areas (VAT) by using the data (slice number, slice thickness, and slice distance), saved in the Dicom format.

#### 2.3.2. DXA Evaluation

**Figure 4:**
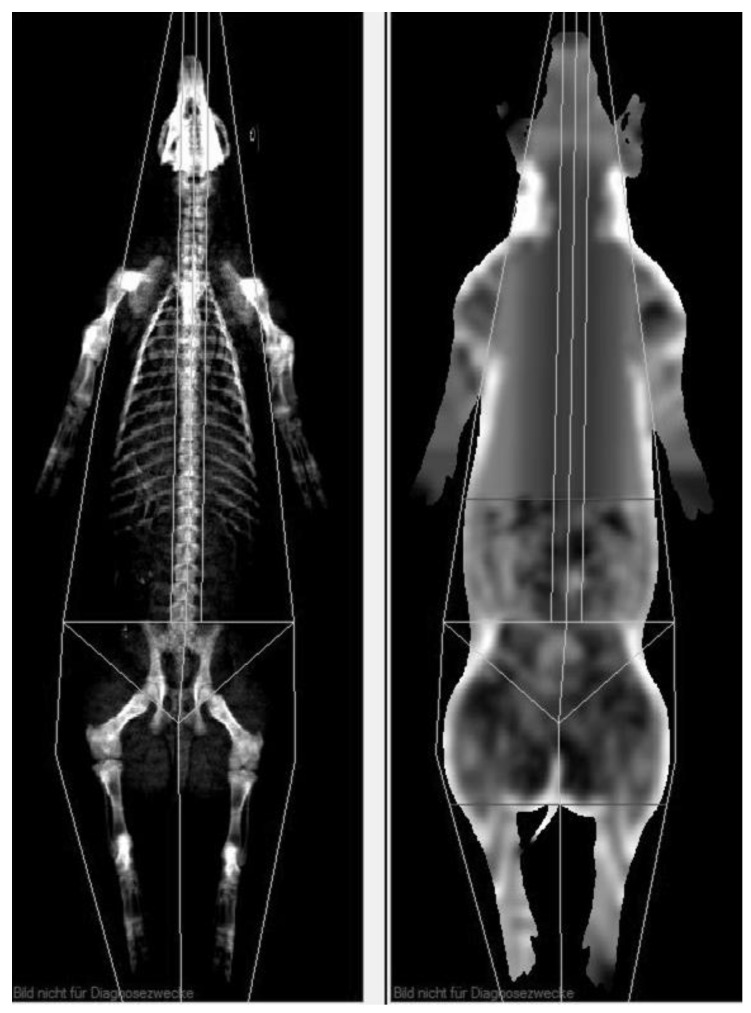
DXA evaluation with enCore software. The uppermost horizontal line (soft tissue image on the right side) defines the level of the origin of the last rib, and the next horizontal line on the top of the iliac crest represents the end of the examined region of VAT.

To get a meaningful comparison of visceral adipose tissue volume between MRI and CoreScan results, the examined body region must be identical. Therefore, the android region was determined individually to encompass the whole region of interest, as shown in Figure 4. The ROI was defined in the “thick” mode, copied for the “standard” mode, and adapted, if necessary, to cover the ROI of the MRI examination. For the study, weight (kg), bone mineral density (g/cm^2^), bone mineral content (g), the amount (g) and percentage of fat tissue and lean tissue mass in the android region, and the mass (g) and volume (cm^3^) of the visceral adipose tissue were measured.

#### 2.3.3. Statistical Analysis

A variance analysis was performed by using a mixed model procedure and restricted maximum likelihood (REML) estimation with the software SAS 9.3 (SAS Institute Inc., Cary, North Carolina, USA). The significance level was set to *p* < 0.05. Genetic origin and the sex of the animals were defined as fixed effects and the date of scan as a random effect. In addition, a linear regression analysis was performed. The coefficient of determination (R^2^) in combination with the standard error of estimation (identical with RMSE = root mean squares error) serves as a measure of the quality of the fit of the regression equation between dependent and independent variables. Furthermore, a Bland–Altman analysis [21] was performed to characterize differences between DXA and MRI VAT measurements over the observed range of VAT volumes.

## 3. Results

### 3.1. Internal DXA Measurement Results

**Figure 5:**
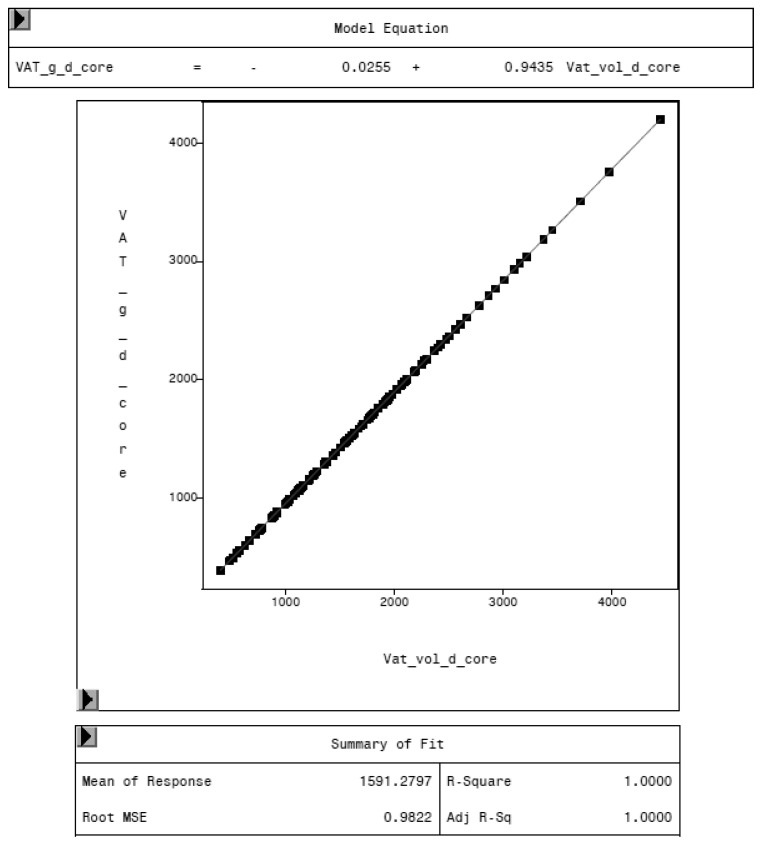
Relationship between VAT mass (VAT_g_d_core, in g) and VAT volume (VAT_vol_d_core, in cm^3^) in “thick” mode, measured by DXA. The model equation shows a density of 0.9435 g/cm^3^ for VAT.

**Figure 6:**
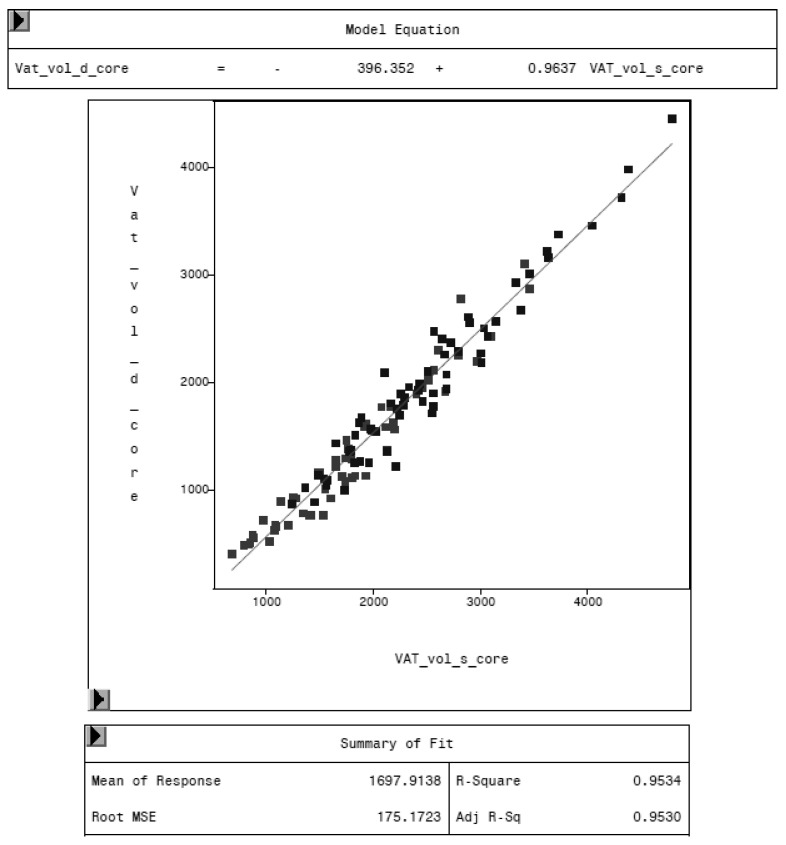
Relationship between VAT volume in “thick” mode (VAT_vol_d_core, in cm^3^) and VAT volume in “standard” mode (VAT_vol_s_core, in cm^3^) measured by DXA. “Standard” mode yields higher values of VAT volume, as the model equation represents.

**Figure 7:**
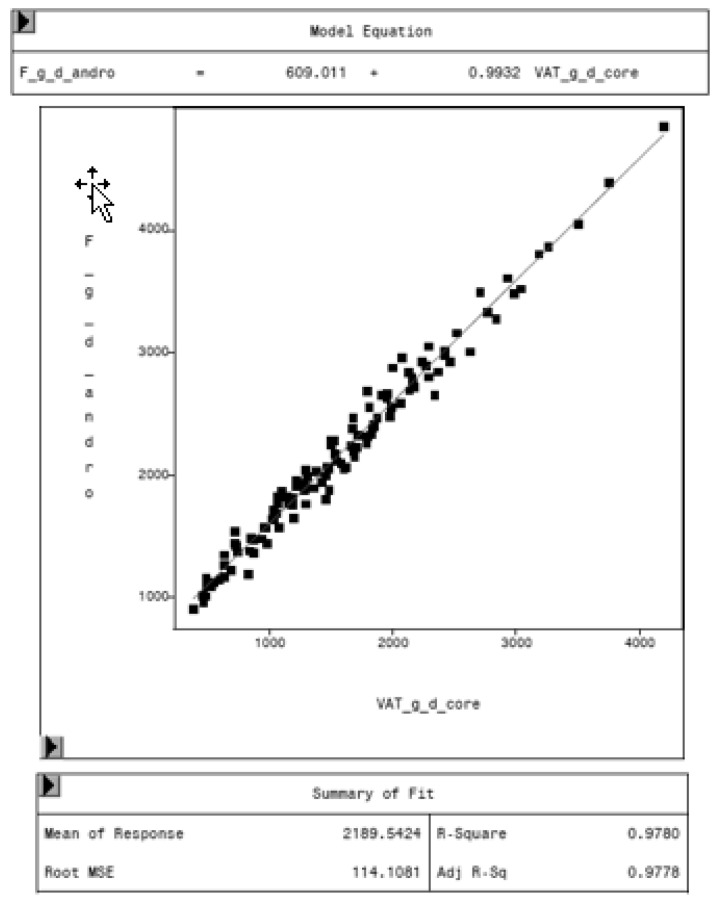
Relationship between fat mass in the android region (F_g_d_andro, in g) and VAT mass (VAT_g_d_core, in g) in “thick” mode, measured by DXA. Regression analysis shows the difference in total fat mass in the android region and VAT. The difference represents the mass of subcutaneous adipose tissue in the average, around 609 g.

To evaluate the CoreScan feature, internal DXA measurement results were compared within each mode and between the two modes, “thick” and “standard”. Within the modes, the regression coefficient between mass (g) and volume (cm^3^) of VAT is expectedly 1.0 and the model equation provides the density estimate of 0.9435 g/cm^3^ for VAT, as shown in Figure 5 (e.g., for thick mode). A very strong relationship (R^2^ = 0.95, RMSE = 175 cm^3^) was found between the volumes of VAT for the two DXA modes. CoreScan obtained higher values for the “standard” compared to the “thick” mode, as shown in Table 3 and Figure 6. Similar relationships can be seen for the amount (g) and the percentage of whole-body fat tissue. Within the android region, the fat mass was expectedly higher than the amount of VAT, because the android region additionally includes the subcutaneous adipose tissue (SAT) (R^2^ = 0.98, RMSE = 114 cm^3^), as shown in Figure 7.

### 3.2. Comparison of MRI and DXA Results

**Figure 8:**
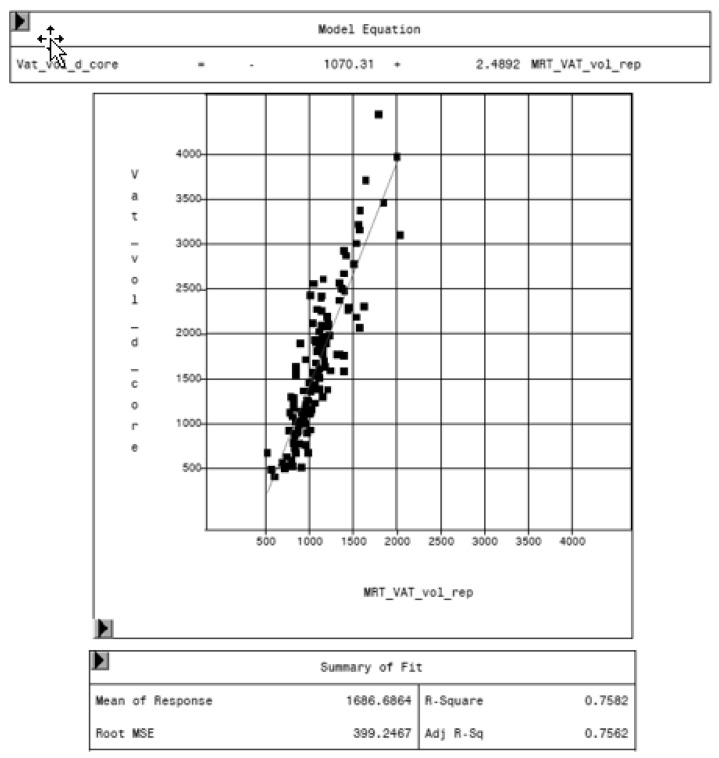
Relationship between volume of VAT measured by MRI (MRT_VAT_vol_rep, in cm^3^) and DXA “thick” mode (VAT_vol_d_core, in cm^3^).

**Figure 9:**
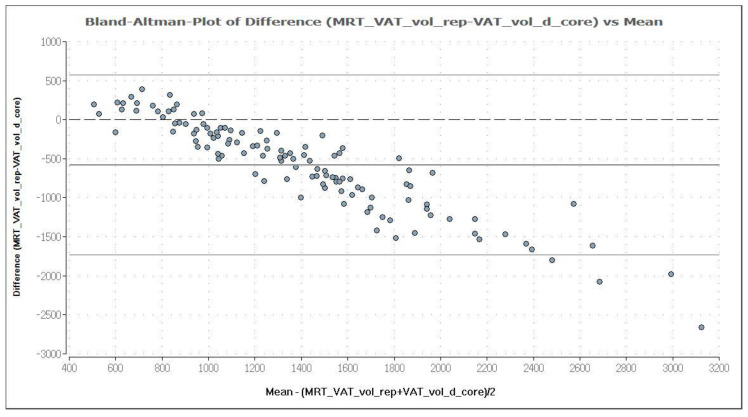
Bland–Altman plot of VAT volume (in cm^3^) measured by DXA in “thick” mode (VAT_vol_d_core) and MRI (MRT_VAT_vol_rep). The mean and the difference of both methods are demonstrated in a Bland–Altman plot. The analysis shows a negative association, which means an increasing deviation of DXA measurement results compared to MRI with increasing levels of VAT. DXA overestimates VAT systematically, compared with MRI, providing a mean difference of −579.1 cm^3^, which is represented by the fat (dark) solid line. The upper and lower solid (light grey) lines represent the limits of agreement (95%), and the broken line represents the line of zero differences.

**Figure 10:**
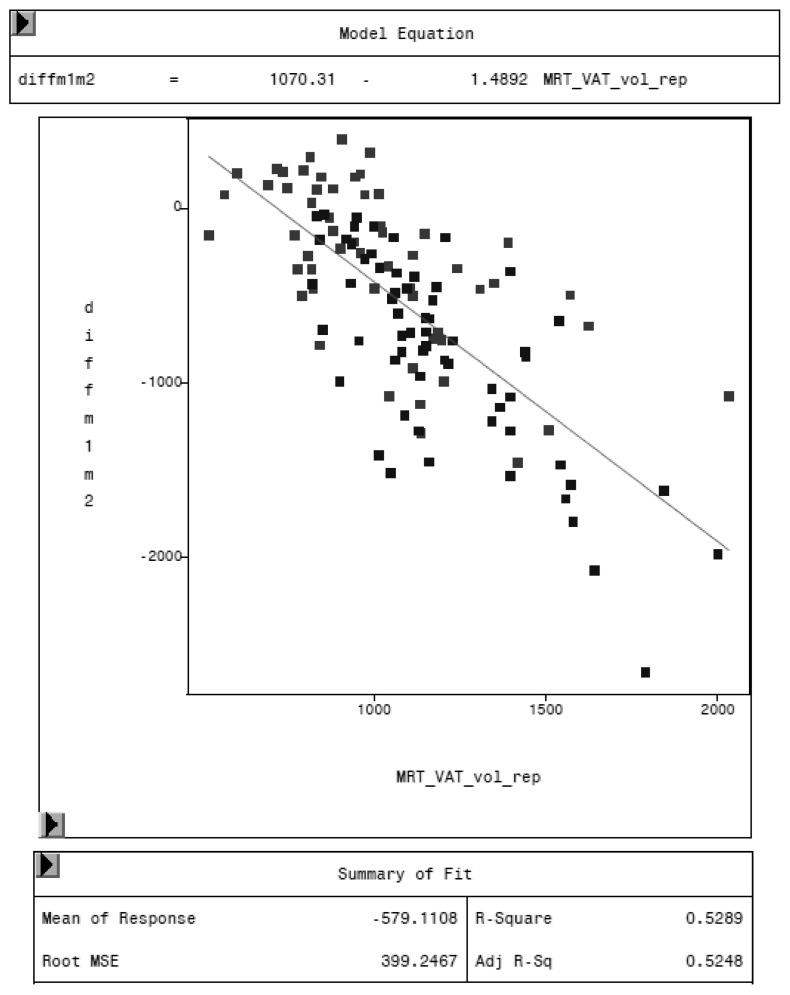
Relationship between the difference of both methods (MRI and DXA-diffm1m2) and the VAT volume measured by MRI (MRT_VAT_vol_rep) in cm^3^.

MRI was used as reference method to evaluate the potential of the CoreScan feature in pigs. In Table 3, the arithmetic mean values of VAT volume, measured by DXA in both modes and by MRI are shown. A close linear relationship can be observed by comparing these VAT volumes (“thick”: R^2^ = 0.76; “standard”: R^2^ = 0.71), as shown in Figure 8 and Table 4. In the Bland–Altman analysis, a negative association was observed between the mean and the difference of both methods, as shown in Figure 9. The average volume calculated by CoreScan (“thick” and “standard”), however, is about 1.5- to 2-times greater than the average volume of the MRI scans, as shown in Table 3 and Figure 10 (only DXA “thick” mode). Therefore, DXA (here only “thick” mode) with the CoreScan feature tends to overestimate the VAT volume compared with MRI, providing a mean difference of −579.1 cm^3^ (95% confidence interval between −684.7 cm^3^ and –473.5 cm^3^, *p* < 0.001). The deviation between DXA and MRI measurement results increases with increasing levels of VAT and is shown in Figure 10. The difference in both methods rises with a slope of 1.5 (1.498) cm^3^ per cubic centimeter more VAT, measured by MRI.

### 3.3. Relationship of VAT, Weight, and Age

**Figure 11:**
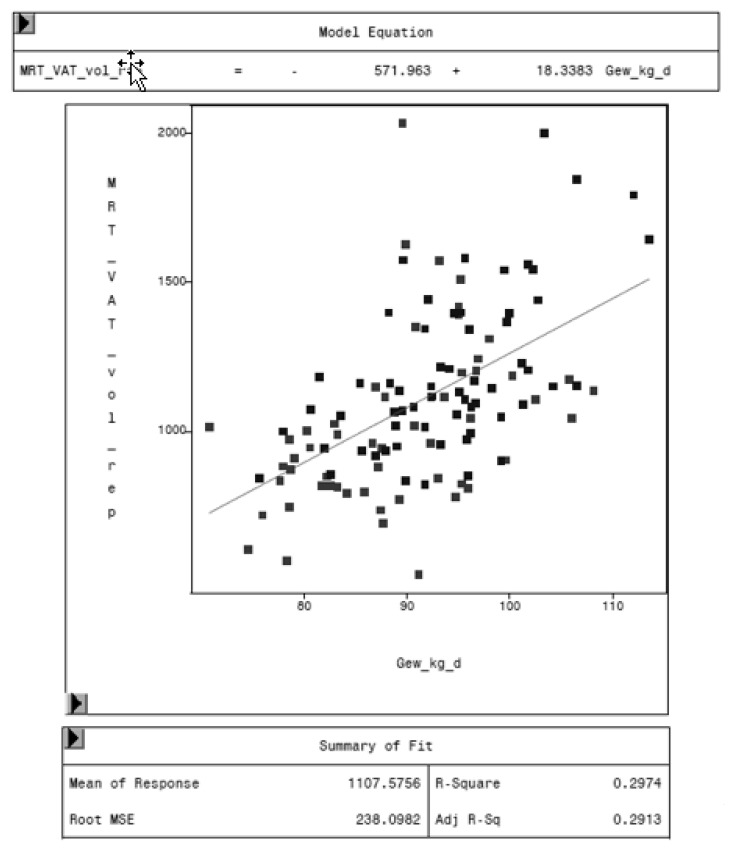
Relationship between VAT volume measured by MRI (MRT_VAT_vol_rep, in cm^3^) and body weight (Gew_kg_d, in kg) of the animal.

The regression analysis between VAT volume of MRI and the weight of the animal shows an increasing VAT with increasing weights. One kilogram more weight leads to 18.3 cm^3^ more visceral adipose tissue, as shown in Figure 11.

Despite the small age range, differences were found in volumes of visceral adipose tissue. The regression analysis shows greater volumes in older pigs for MRI and DXA. Volumes of visceral adipose tissue increase by 34 cm^3^ (MRI) and 109 cm^3^ (DXA) per day of life, as shown in Table 5.

### 3.4. Variation in Fat Mass and VAT Volume by Sex and Genetic Origin

The variance analysis of fat mass in the android region shows significant differences between sex groups (*p* = 0.0002) and both crossbred lines (*p* = 0.0243). MHF1 pigs have, on average, 319.6 g more fat in the android region than MHF2 individuals. We also observed a higher fat mass in castrated males compared to female pigs (+452.2 g). The mass and percentage of total body fat differs by sex and crossbred line in the same way as fat in the android region, with MHF1 animals and castrated pigs showing higher values than MHF2 individuals or females, respectively, although, the total body fat mass did not differ significantly (*p* = 0.0504 or *p* = 0.0787) between crossbred lines, as shown in Table 6.

The volume of VAT measured by DXA (“standard”, “thick”) and MRI differs significantly (*p* < 0.05) for sex and genetic origin. Castrated male pigs, and also the first generation of crossbreds (MHF1), have a larger VAT volume, as shown in Table 6.

### 3.5. Variation in Weight by Sex and Genetic Origin

Weight differed not significantly between both crossbred generations (*p* = 0.1268). As in the previous results, a sex-related difference was observed with castrated males, achieving higher values. On average, a castrated male pig weighed 3.9 kg more than a female individual, as shown in Table 6.

## 4. Discussion

MRI is considered as the gold standard of VAT determination in humans [22,23] and is, together with CT, the in vivo method of choice for directly quantifying the distribution of adipose tissue [22,24,25,26]. MRI is characterized by a high accuracy, no radiation exposure, and shows a good reproducibility with a coefficient of variation for repeated VAT measurements of approximately 9% to 18%, as Shen et al. summarized in their review. However, MRI has significant limitations: limited access; high cost; prolonged scan time [22,24]. MRI causes high investment costs (which increase with the field strength of the magnet), as well as high running costs for the system service, including temperature control and general maintenance. In addition, compared to DXA, an MRI system requires more building space and a Faraday cage surrounding the magnet. The MRI sequences in our study took 12.22 min and 10.27 min for examining the region of the abdomen and pelvis, respectively; with the repositioning of the animal between the sequences. In comparison, the more suitable DXA method (“thick” mode in our case) needs a whole body scan, which takes a maximum of 13.45 min (“standard” only 7.25 min). Besides the prolonged scan time, the evaluation of the MRI images is more time-consuming compared to the DXA evaluation. In our case, the number of slices needed to cover the whole region of the abdomen was about 30. The VAT had to be defined manually for each MRI slice, because an automatic or semi-automatic approach lacked sufficient anatomic accuracy. The DXA enCore software automatically suggests lines for specific body regions. These lines had to be only slightly adjusted in our study in order to cover the identical body region, as in the MRI evaluation. The DXA evaluation of VAT per animal usually took less than 5 min, while the MRI VAT evaluation took up to 5 h per animal. Furthermore, MRI specialists are needed both for the examination and for the image evaluation. In the DXA enCore software, no settings need to be especially defined, as is essential for MRI (due to different sequences). Since our study shows the ability of the DXA CoreScan feature to provide VAT estimates in pigs closely related to MRI, the CoreScan mode can be a fast, relatively simple, and a low-cost option to detect the volume of visceral adipose tissue, compared with MRI.

In Contrast to MRI or CT, however, DXA only measures two-dimensionally. Therefore, subcutaneous and visceral adipose tissue cannot be distinguished directly. The amount of VAT can only be estimated [27,28]. Consequently, VAT depends on the amount of SAT, and an inaccuracy of detecting SAT will lead to biased results of VAT. This might be one source of error explaining the overestimation of VAT measured by DXA that was found in our study. Further studies of quantifying the SAT in the android region may help to confirm or exclude this source of error. Fourman et al. found an overestimation of SAT combined with an underestimation of VAT compared to a single image CT in humans by using a Hologic Horizon A DXA scanner [29].

In pigs, MRI and DXA (including pure bone mineral analysis [30,31]) are also used for body composition analyses and achieve acceptable results compared to chemical analyses or dissection [32,33,34,35,36]. Our study shows that DXA overestimates VAT compared to MRI and the deviation rises with increasing VAT levels in accordance with other porcine and human studies, where DXA tends to overestimate the amount of fat [33,37,38,39,40]. Examinations in pigs related to VAT by MRI and DXA, however, have not been reported yet. Mohammad et al. compared VAT values from enCore software with MRI results, using identical anatomical regions for both techniques in Kuwaiti men and women, and found an overestimation of 79.7 cm^3^ (95% limits of agreement, −767 cm^3^ to 963 cm^3^) in men and 46.8 cm^3^ (95% limits of agreement, −482 cm^3^ to 866 cm^3^) in women and an increasing imprecision with increasing VAT levels [37]. In pigs, Mitchell et al. found that DXA (Lunar DPX-L) tends to overestimate the total body fat content in pigs that have more than 20% body fat and underestimates the fat content in leaner pigs, compared to chemical analysis [33].

The overestimation might be explained by the fact that scanning too quickly or with insufficient X-ray source current, depending on body thickness, a degradation of the image quality would result by “starving” each pixel of X-ray photons [41]. Therefore, especially in animals with a larger body thickness, the X-rays are attenuated to a greater extend and may result in an inaccuracy of the results. To compensate, the influence of body thickness, the enCore software offers different modes related to different body thicknesses (“standard”: 15–25 cm; “thick”: > 25 cm) combined with a prolonged scan time (more X-ray photons per scan area) in “thick” mode, leading to a higher absorbed dose (6 µGy in “thick” mode vs. 3 µGy in “standard” mode). With the “thick” mode, we measured VAT values closer to the MRI results than with the “standard” mode, which confirms the influence of X-ray photon intensity on the 2D image quality and underlying X-ray attenuation coefficients (R values).

Lukaski et al. examined the effect of body thickness on the difference in chemical and DXA analysis results using a Hologic 2000W scanner. The total errors in the determination of DXA body composition variables were similar with body thicknesses greater and less than 24 cm, and no effect was associated with the tissue thickness and estimation of fat [42]. This observation is in contrast to our study and to others, where tissue thicknesses of 20–25 cm were associated with an overestimation of fat in and ex vivo [43,44]. The results of our study show an increasing overestimation of VAT measured by DXA compared to MRI results with increasing levels of VAT. To compensate the overestimation of DXA, the VAT values need to be bias corrected, according to the regression equations shown in Table 4.

In general, the comparison of studies measuring VAT or total fat in the android region is difficult for several reasons. Firstly, it is essential to examine the same anatomical region with both applied methods. In CoreScan, the android region is defined as 20% of the distance between the top of the iliac crest and the base of the skull and, therefore, the region varies according to the length of the trunk [45]. Using a fixed region in MRI would lead to inaccuracies. For example, Neeland et al. used a single MRI slice at the L2–L3 intervertebral level and compared it with an estimation of VAT mass at the L4–L5 region in DXA [38]. To avoid this source of error, we defined an exact body region for the MR imaging, which starts at the origin of the last rib and reaches to the top of the iliac crest. The android region, by using the DXA enCore software, was adapted to cover this defined region. Secondly, other reasons for the difficulty of comparing studies are: different analytical methods, including multiple or single slice MRI, different equipment and software, such as GE Healthcare/CoreScan or Hologic/InnerCore instruments, and the measured parameters (area or volume). Summarizing our results, DXA “CoreScan” has the ability to estimate VAT in pigs with a close relationship to MRI, but it is biased, according to the Bland–Altman analysis, as shown in Table 3 and Table 4 and Figure 8 and Figure 9.

In humans, the well-known gender-related differences in fat distribution arise, among other things, due to sexual hormones, which have multiple effects on adipose tissue [46]. Various studies found that adipose tissue presents sexual steroid receptors for estrogens, androgens, and progesterone [47,48,49]. The expression of these receptors varies by depot and gender. In intra-abdominal preadipocytes, the number of androgen binding sites is higher than in subcutaneous fat depots in males and females [48]. In males, the density of estrogen receptors in visceral adipose tissue is lower than in subcutaneous depots, and the binding capacity in SAT is also higher compared to VAT [50,51]. Another fact, which underlines the effect of sexual hormones is that sexual maturity influences total body fat and subcutaneous and visceral adipose tissue [22].

For translational medicine, it is important to characterize the animal model by identifying the potentials and limitations. Especially in the field of obesity research (e.g., metabolic syndrome), gender-associated differences are an evolving issue in human medicine and should be addressed in the animal model. Therefore, it is essential to consider the discrepancies in sex hormone patterns between pigs and humans [52,53]. Several studies examined female and male (intact) minipigs and their ability to be a model for metabolic syndrome or in general for obesity research. Females had more total body fat, including more visceral fat. Metabolic abnormalities were more severe compared to intact male minipigs, which might be explained by the higher amount of fat. In conclusion, it was postulated that female minipigs might better display the obesity-related consequences in humans. Different hormone concentrations could be seen as a possible cause for the observed differences between male and female (mini-)pigs. Male (intact) minipigs have extremely high concentrations of testosterone, while their concentration of estradiol is even higher than in females before and after sexual maturity [52,53]. This phenomenon has also been reported for crossbreeds. Crossbred boars showed higher estradiol concentrations than crossbred females, already by the 98th day of life [54]. In general, the high concentration of testosterone and estradiol protects male minipigs from obesity and depositing fat (including VAT) at a high rate. Comparing the hormone concentrations to the situation in humans, the estradiol in female minipigs displays the concentration in post-menopausal women or men, while the concentration of estradiol in intact male minipigs is more similar to the concentration in pre-menopausal women, but is still even lower than in pre-menopausal women [52,53]. In our study, castrated male pigs and female pigs were examined shortly before sexual maturity. The observed amounts of VAT, which were higher in our castrated males, were similar to the human situation between intact men and women. However, they also showed a higher total fat content, which is in contrast to humans, where women have greater amounts of total body fat than intact men. Christoffersen et al. studied the influence of castration in Göttinger minipigs and found a complete disappearance of circulating testosterone and estradiol, resulting in an increased food intake, increased body weight, and also an increased body fat content. Already at 10 to 11 weeks after castration, the pigs were more insulin resistant, showed higher glucose intolerance and hyperglucagonemia. In general, low testosterone and estradiol concentrations are predictive for metabolic syndrome [55]. Therefore, castrated male pigs may be even more suitable to examine obesity-related consequences, because they achieved higher levels of VAT compared to females. In addition, castrated male pigs with a complete absence of sexual steroids might be used as a neutral basis for studies on the effects of hormones on obesity, VAT, and their related consequences after the application of steroids. The use of defined steroid concentrations offers the opportunity to readjust the situation in humans as accurately as possible with a simultaneous unchanged gene situation.

Similar findings are described in pigs, where the proportion of fat increases in the carcasses of older and heavier pigs [56,57]. In accordance with other studies, this study shows an increase in VAT of 18.3 cm^3^ per kilogram weight in MRI, as shown in Figure 11. Mohrmann et al. found the highest accumulation of fat in animals with a weight range of 90-120 kg, whereas Giles et al. described a smaller range, 80–100 kg [58,59]. Consequently, the pigs in our study were in the weight range of highest fat accumulation and have the potential to be an appropriate model for VAT examination. A study of fat deposition in pigs by CT found an increasing proportion of abdominal fat in total fat with increasing live weight [60].

As described previously, the amount of fat in humans increases with age. In our study, pigs at a relatively young age of 5 months were examined. Already at this time, increasing fat contents were found. Deeper insights into the development of fat could be reached by studying the pigs at several time points and at later stages of age. However, the use of MRI and DXA is limited by the size and weight of the object under investigation. In addition, for research purposes, a preferably early examination of the animals is of interest to save costs and time. Koopmans et al. recorded in their study a weight of 70 kg with 1.2 kg VAT for obese minipigs and compared this pig model to humans. In humans, one kilogram of VAT is already considered as a health threat, usually present at body weights above 100 kg. Furthermore, it was postulated that it takes a time period of five to six months to develop the obese-metabolic-syndrome phenotype [61]. The pigs in our study are at this age and achieved VAT values of more than one kilogram (1.8 kg VAT in castrated males, 1.2 kg in females). Therefore, castrated male pigs are also a suitable model for obesity research.

In pigs, the influence of sex on body and/or carcass composition is widely investigated. The findings of this study, where castrated males have higher amounts of total body fat and also of VAT compared to females, are in agreement with other studies. Carcasses of boars are characterized by the highest lean meat content, combined with a low fat content, followed by gilts. Barrows (castrated male pigs) tend to have fatter carcasses with the lowest lean meat content [57,62]. The results of our study show that DXA has the ability to measure these sex-related differences of total body fat and VAT.

Besides sex, age, and weight, fat deposition is also influenced by genetic background. Body composition in pigs is polygenically controlled, and a large number of genes and markers associated with variations in fat and lean mass content have been identified [14,63,64]. Quantitative trait loci (QTL) should help to localize genes that—besides environmental effects—control specific phenotypes. The database PigQTLdb offers an extensive summary of published QTL information and, so far (as of 26.03.2020), 3138 QTL are associated with the trait “fatness” [65,66]. Rothammer et al. found, in a genome-wide QTL mapping study, 41 significant associations with the percentage of fat, measured by using a whole-body DXA scan on 13 chromosomes in pigs, and hypothesized, for example, the effect of the candidate gene *ZNF608* on fat mass [14]. In humans, the association of *ZNF608* and body mass index has likewise been reported [67,68]. In a recent study, Rothammer et al. examined the effect of regional body composition traits on QTL results. They compared the results of the QTL-mapping analysis using a whole-body DXA scan of 2014 with the findings of QTL in regional DXA analyses (e.g., in the region of the abdomen). Overall, it can be concluded that a whole-body DXA scan provides reliable and substantial results, but specific regional analyses will provide additional knowledge of locally active QTL. For example, seven QTL, that can be associated with possible candidate genes, have been found only in the region of the abdomen, and did not show up in the whole-body QTL analysis. Three of these seven QTL are associated with the phenotype of fat, and possible candidate genes are: polypeptide N-acetylgalactosaminyltransferase 17 (*GALNT17*, previous symbol: *WBSCR17,* chromosome 3); abhydrolase domain containing 6 (*ABHD6*, chromosome 13); pyruvate dehydrogenase E1 subunit beta (PDHB, chromosome 13) [69]. In mutant mice, *WBSCR17* is associated with a decrease in lean body mass. A selective knockdown of *ABHD6* protects mice from high-fat-induced obesity [70,71], while *PDHB* on chromosome 13 has been described in cattle and pigs and is a candidate gene for intramuscular fat deposition [72,73]. It can be hypothesized that the effects of QTL, which are found only by analyzing a specific body region, are predominantly local [69]. Therefore, the regional analyses of fat, and especially of VAT, conducted in our study might provide extra value for genomic analyses. In addition, the simultaneous occurrence of genetic and phenotypic differences is essential for marker-based mapping of QTL and, therefore, the use of different breeds in the initial generation will lead to a high variance in F2 generation [64]. For this reason, four conventional breeds are used in our study for phenotyping in the multiple F1 and F2 crossbreeding generations to create a basis for genomic analyses. Scholz et al. estimate the additive genetic variance components to account for 45–60% of the phenotypic variation of body composition traits [15,63]. A study by Kogelman et al. determines a higher heritability of DXA lean mass of 0.71 compared to 0.43 for DXA fat mass in a F2 population with Göttinger minipigs [74]. In addition to the heritability (proportion of additive genetic variance on phenotypic variance), heterosis effects in crossbred lines may be associated with a variation in body composition. The influence of heterosis is greatest in the F1 generation and declines in further generations. Therefore, especially F1 crossbreds are used for fattening and reproduction, due to their superiority [75,76]. Carcasses of F1 crossbreds are characterized by higher weights, more lean tissue mass, but also higher fat mass [77,78]. In a study by Müller et al., traits such as growth, carcass, and meat quality were examined in wild boars, Meishan and Pietran pigs, and their crossbreeds [78]. All crossbreds showed a higher amount of abdominal fat weight in the F1 compared to the F2 generation, which underlines the results of our study, where F1 pigs have higher amounts of total body fat, fat in the android region, and also more VAT, as shown in Table 6.

Our study dealt with the ability of the DXA CoreScan feature to provide VAT estimates in pigs as a model for human research. The results support the hypothesis that DXA and the CoreScan feature might be applicable to a wider range of species for fat measurements for translational research.

## 5. Conclusions

Our study shows the ability of the DXA CoreScan feature to provide VAT estimates in pigs, but needs a bias correction if MRI VAT is seen as the reference method. The low-cost measuring method described here is more easily accessible and has shorter examination times than MRI, and leads to less exposure to ionic radiation than CT. In addition, the variance analysis of the DXA results underlines the known genetic- and sex-related differences in body composition in pigs, where castrated males and the first crossbred generation tend to have higher amounts of total body fat and visceral fat depots. Further studies into distinct fat depots, especially SAT in the android region, will help to evaluate the CoreScan feature in more detail. A combination of describing the phenotypic variance by in vivo techniques (MRI, DXA) with genomic analyses will help to identify more genes related to body composition traits. These findings will have an impact on human health by contributing new prospects to the field of obesity research.

## Figures and Tables

**Table 1:** Animal sample.

	Number	Age (days)		Weight (kg)	
		Mean	Standard Deviation	Mean	Standard Deviation
Castrated males	63	147.4	3.5	93.9	7.6
Females	57	146.9	3.1	89.0	8.4
MHF1	58	147.8	3.5	93.0	8.1
MHF2	62	146.5	3.1	90.3	8.5

**Table 2:** MRI protocol of both T1-weighted sequences—“ViscFat” and “Ham” sequences.

	ViscFat Sequence	Ham Sequence
pixel size (mm x mm)	1.9 × 1.6	1.9 × 1.6
examination time (min)	12.22	10.27
signal-to-noise ratio	1.00	1.00
repetition time (TR) (ms)	441	370
echo time (TE) (ms)	18	18
slice number	30	20
slice thickness (mm)	6	6
Acquisition	axial	axial
distance factor (%)	20	20
matrix size	211 × 250	211 × 250
field of view (FoV) (mm)	400	400

**Table 3:** Arithmetic means and standard deviations of VAT volume measured by DXA for “thick” and “standard” modes, and by MRI.

VAT Volume (cm^3^)			
	DXA “Thick”	DXA “Standard”	MRI
Arithmetic mean	1686.69	2173.08	1108.33
Standard deviation	805.08	815.17	283.76

**Table 4:** Relationship between volume of VAT measured by MRI (MRT_VAT_vol_rep) and DXA “thick” mode and DXA “standard” mode.

	DXA VAT Volume “Thick” (cm^3^)	DXA VAT Volume “Standard” (cm^3^)
Model equation	−1070.31 + 2.4892 MRT_VAT_vol_rep	−537.384 + 2.4368 MRT_VAT_vol_rep
Root MSE	399.2	443.4
Adj. R-Sq	0.756	0.707
Pr > t	<0.0001	<0.0001

Root MSE = root mean square error; Adj. R-Sq = adjusted R-square; Pr > t = defines the significance level.

**Table 5:** Relationship between MRI VAT volume and DXA VAT volume in “thick” mode and the age of the pig.

	MRI VAT Volume (cm^3^)	DXA VAT Volume “Thick” (cm^3^)
Model equation	−3908.40 + 34.0868 age (days)	−14,353.7 + 109.005 age (days)
Root MSE	261.9	724.9
Adj. R-Sq	0.16	0.20
Pr > t	<0.0001	< 0.0001

Root MSE = root mean square error; Adj, R-Sq = adjusted R-square; Pr > t = defines the significance level.

**Table 6:** Least squares means (LSM), standard errors of estimation (SEE), and significance levels of examined parameters.

			Sex			Genetic Origin	
				Castrated Males	Females	MHF1	MHF2
DXA	Mode “Thick”	Gew_kg_d [kg]	LSM	93.59	89.69	93.19	90.09
			SEE	1.33	1.39	1.57	1.50
			Pr > t	0.0079		0.1268	
		F_g_d_ges [g]	LSM	15,092	12,554	14,532	13,114
			SEE	466	492	533	507
			Pr > t	<0.0001		0.0504	
		F_proz_d_ges [%]	LSM	16.35	14.14	15.86	14.63
			SEE	0.40	0.43	0.46	0.44
			Pr > t	<0.0001		0.0498	
		F_g_d_andro [g]	LSM	2409	1957	2343	2023
			SEE	91	96	104	99
			Pr > t	0.0002		0.0243	
		VAT_g_d_core [g]	LSM	1868	1290	1741	1416
			SEE	89	94	103	98
			Pr > t	<0.0001		0.0200	
		VAT_vol_d_core [cm^3^]	LSM	1979.53	1367.33	1845.77	1501.88
			SEE	94.82	100.01	108.99	103.75
			Pr > t	<0.0001		0.0200	
	Mode “Standard”	F_g_s_ges [g]	LSM	16,164	13,677	15,537	14,304
			SEE	454	481	511	486
			Pr > t	<0.0001		0.0787	
		F_proz_s_ges [%]	LSM	17.56	15.46	17.01	16.02
			SEE	0.37	0.39	0.41	0.39
			Pr > t	<0.0001		0.0830	
		F_g_s_andro [g]	LSM	2604	2164	2531	2237
			SEE	86	91	96	91
			Pr > t	0.0003		0.0272	
		VAT_g_s_core [g]	LSM	2335	1744	2182	1897
			SEE	84	89	92	88
			Pr > t	<0.0001		0.0269	
		VAT_vol_s_core [cm^3^]	LSM	2475.38	1848.46	2312.55	2011.30
			SEE	88.62	93.93	97.66	92.74
			Pr > t	<0.0001		0.0270	
MRI	MRT_VAT_vol_rep [cm^3^]		LSM	1188.83	1016.65	1196.40	1009.08
			SEE	47.48	49.08	56.44	53.86
			Pr > t	0.0001		0.0074	

Gew_kg_d [kg]: Body weight; F_g_d_ges [g]: Total fat mass in mode “Thick”; F_proz_d_ges [%]: Percentage of total fat mass in mode “Thick”; F_g_d_andro [g]: Android fat mass in mode “Thick”; VAT_g_d_core [g]: VAT mass in mode “Thick”; VAT_vol_d_core [cm^3^]: VAT volume in mode “Thick”; F_g_s_ges [g]: Body weight; F_proz_s_ges [%]: Total fat mass in mode “Standard”; F_g_s_andro [g]: Percentage of total fat mass in mode “Standard”; VAT_g_s_core [g]: Android fat mass in mode “Standard”; VAT_vol_s_core [cm^3^]: VAT mass in mode “Standard”; MRT_VAT_vol_rep [cm^3^]: MRI VAT volume.
